# Study design of a stepped wedge cluster randomized controlled trial to evaluate the effect of a locally tailored approach for preconception care – the APROPOS-II study

**DOI:** 10.1186/s12889-020-8329-1

**Published:** 2020-02-14

**Authors:** Veronique Y. F. Maas, Maria P. H. Koster, Erwin Ista, Kim L. H. Vanden Auweele, Renate W. A. de Bie, Denhard J. de Smit, Bianca C. Visser, Elsbeth H. van Vliet-Lachotzki, Arie Franx, Marjolein Poels

**Affiliations:** 1 Department of Obstetrics and Gynaecology, University Medical Centre Rotterdam, Rotterdam, Doctor Molewaterplein 40, 3015 GD the Netherlands; 2 Department of Internal Medicine - Nursing Science, Erasmus MC, University Medical Centre Rotterdam, Rotterdam, Doctor Molewaterplein 40, 3015 GD the Netherlands; 3 HELLP foundation, Zwolle, Postbus 40126, 8004 DC the Netherlands; 4 Department of Obstetrics, Diakonessenhuis hospital, Utrecht, Bosboomstraat 1, 3582 KE the Netherlands; 5 MediClara Projects, Baambrugge, Prinses Beatrixstraat 7, 1396 KD the Netherlands; 6 Geboorte Concortium Midden Nederland (GCMN), Utrecht, Oudlaan 4, 3515 GA the Netherlands; 7 Dutch Genetic Alliance, VSOP, Utrecht, Koninginnelaan 23, 3762 DA Soest, the Netherlands; 8 Research agency Care2Research, Amsterdam, Mattenbiesstraat 133, 1087 GC the Netherlands

**Keywords:** Preconception care, Pregnancy planning, Maternity care, Behavioural change, Health behaviour, Pregnancy planning, Health promotion, Healthcare providers

## Abstract

**Background:**

In a previous feasibility study (APROPOS) in a single municipality of the Netherlands, we showed that a locally tailored preconception care (PCC) approach has the potential to positively affect preconceptional lifestyle behaviours. Therefore, we designed a second study (APROPOS-II) to obtain a more robust body of evidence: a larger group of respondents, more municipalities, randomization, and a more comprehensive set of (clinical) outcomes. The aim of this study is to assess the effectiveness and the implementation process of a local PCC-approach on preconceptional lifestyle behaviours, health outcomes and the reach of PCC among prospective parents and healthcare providers.

**Methods:**

This study is an effectiveness-implementation hybrid type 2 trial. This involves a stepped-wedge cluster randomized controlled trial design aiming to include over 2000 future parents from six municipalities in the Netherlands. The intervention has a dual-track approach, focusing on both the uptake of PCC among future parents and the provision of PCC by healthcare providers. The PCC-approach consists of 1) a social marketing strategy directed towards prospective parent(s) and 2) a local care pathway to improve interdisciplinary collaboration. Data will be collected before and after the introduction of the intervention through questionnaires and medical records in each of the participating municipalities. The primary outcome of this study is change in lifestyle behaviours (e.g. folic acid use, smoking and alcohol use). Secondary outcomes are pregnancy outcomes (e.g. miscarriage, preterm birth, gestational diabetes) and the uptake of PCC. Moreover, a process evaluation will be performed, providing information on the efficacy, feasibility, barriers and facilitators regarding the implementation of the intervention.

**Discussion:**

The APROPOS-II study introduces a locally tailored PCC-approach in six municipalities in the Netherlands that will contribute to the body of evidence regarding the effectiveness of PCC and its implementation. If this intervention has a positive effect on lifestyle behaviour changes, leading to improved pregnancy outcomes and the future health of prospective parents and their offspring, it could subsequently be upscaled to (inter)national implementation.

**Trial registration:**

Dutch Trial register: NL7784 (Registered June 6th, 2019).

## Background

Despite major advances in clinical research and medical technology, the prevalence of adverse maternal and neonatal health outcomes, such as pre-eclampsia and preterm birth, have only moderately decreased over the past decade [1]. As the first few weeks of pregnancy are crucial for gametogenesis, organogenesis and placental development, there is growing evidence that exposure to unhealthy lifestyle behaviours before or during pregnancy (such as alcohol consumption, smoking, physical inactivity, excessive weight gain, obesity and poor nutrition), often affect the future health of mothers, their offspring and future generations [2–4]. In the Netherlands, 85% of all pregnancies are planned pregnancies [5]. However, planning a pregnancy is not always accompanied by appropriate preparation for pregnancy. As such, only half of all pregnant women use folic acid in the correct dose for the correct amount of time and more than 60% of all women with a wish to conceive still use alcohol [5]. A recent study among future fathers showed that the majority of them took no action to improve their lifestyle behaviours before conception [6]. Therefore, it is important to improve awareness among prospective parents regarding (unhealthy) preconceptional lifestyle behaviours and its effect on reproductive outcomes and to encourage prospective parents to actively prepare for pregnancy.

One way to actively prepare for pregnancy is by using preconception care (PCC). PCC is defined as “a set of interventions that aim to identify and modify medical, behavioural and social risks to a woman’s health or pregnancy outcome through prevention and management” [7]. Despite a growing body of evidence showing that PCC can increase the health and well-being of prospective parents, the uptake of PCC-consults remains remarkably low [8]. Even more cause for concern is that vulnerable women, who often have multiple unhealthy lifestyle behaviours, are specifically hard to reach [9]. Previous studies have shown that barriers for the use of PCC are determined by the presence of preconditions, women’s beliefs, perceptions and experiences, given the limited availability and the inadequate infrastructure in which PCC is provided [10, 11]. Previously developed PCC-interventions tend to be clinical and focus on individual-level behaviour change (i.e. counselling women not to engage in risky behaviours) rather than examining social, structural and environmental factors that shape preconception health [12].

A previous feasibility study performed by our group (APROPOS) in one municipality of the Netherlands showed that a locally tailored PCC-approach has the potential to positively affect preconceptional lifestyle behaviours and increases the use of PCC among prospective parents [13]. After being exposed to the intervention, women were more likely to make at least one preconceptional lifestyle behaviour change compared to women who were not exposed to the intervention (adjusted OR 1.56 (95%CI 1.02–2.39)) [13]. However, before this locally tailored intervention can be implemented on an (inter)national level, the feasibility and effectiveness of this intervention needs to be analysed in a larger-scale implementation study. Therefore, we designed a second study (APROPOS-II) with implementation in more municipalities, a larger group of respondents, randomization, and assessment of a more comprehensive set of (clinical) outcomes.

### Aims and objectives

The aim of this study is to assess the effectiveness and the implementation process of a local PCC-approach (i.e. the intervention) on preconceptional lifestyle behaviours, health outcomes and the reach of PCC among prospective parents and healthcare providers. Therefore, we have specified the following objectives:

- To determine the effect of the intervention on preconceptional lifestyle behaviours (e.g. smoking, alcohol use, folic acid use, physical activity) and other risk factors (e.g. body mass index, psychological distress, chronic illness) among women who recently conceived;
- to assess the effect of the intervention on the reach of prospective parents regarding PCC, the uptake of PCC and pregnancy preparation;
- to evaluate the implementation process in order to gain information on the feasibility, barriers and facilitators for the implementation of the local PCC-approach;
- to evaluate the sustainability of the intervention, with a specific focus on the diversity within and between the participating municipalities.

## Methods / design

### Study design

**Fig. 1:**
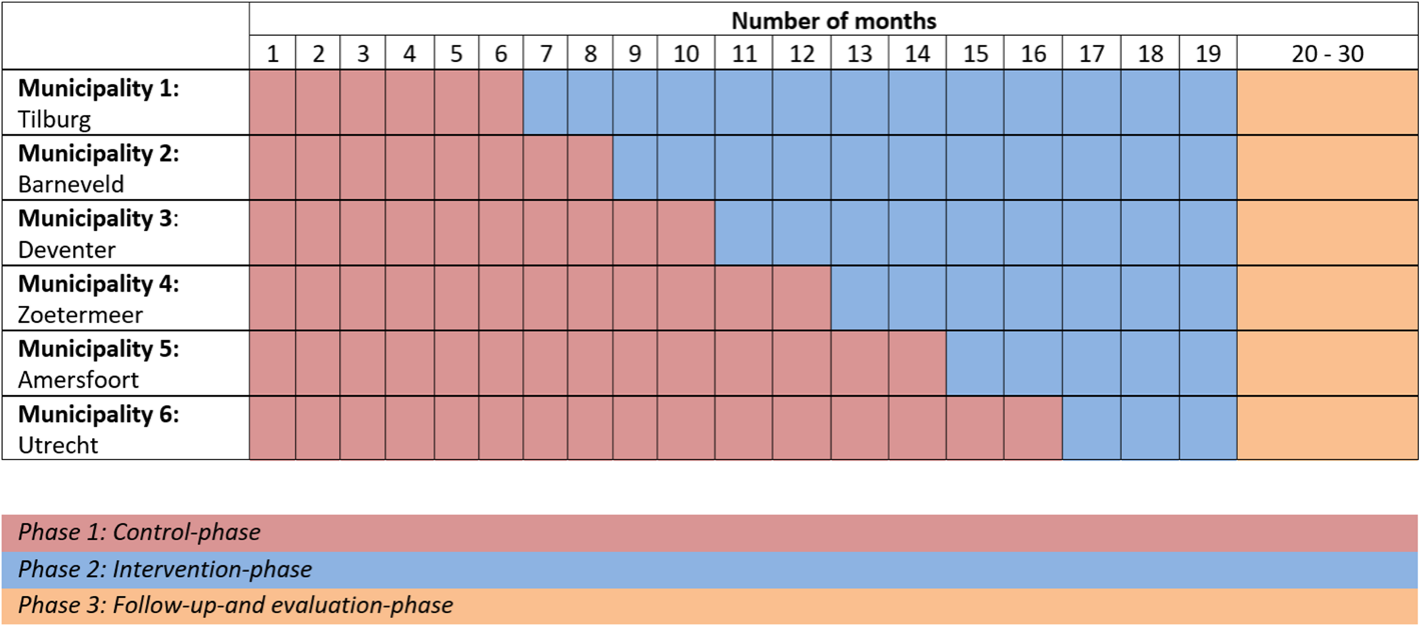
Timeline of the APROPOS-II study. All participating municipalities start with a control-phase, which will last 6–16 months depending on randomization order. After the control phase, the intervention will be implemented stepwise in every municipality

The APROPOS-II study uses a hybrid effectiveness-implementation design [14, 15]. In a stepped-wedge cluster-randomized controlled trial (RCT) we will implement and evaluate the effectiveness of the intervention. Randomization occurs at a cluster-level instead of an individual-level because the intervention has a community-approach in which the entire target population will be exposed to the intervention. All participating municipalities start with a control-phase, which will last 6–16 months depending on randomization order. The total duration of the study is 30 months (Fig. 1). The RE-AIM (reach, effectiveness, adoption, implementation, maintenance) framework will be used to evaluate the intervention and the implementation strategy [16].

### Study population and setting

The study will be conducted in six municipalities distributed throughout the Netherlands (i.e. Amersfoort, Barneveld, Deventer, Tilburg, Utrecht and Zoetermeer). In total, ten community midwifery practices in these municipalities participate in the study. Municipalities were selected for diversity regarding their size (varying from 57,000–211,000 inhabitants), population, health challenges and organization of care. All women above the age of 18 years who adequately master the Dutch, English, Polish or Turkish language are eligible to participate in this study. Men are recruited through their participating partners.

**Fig. 2:**
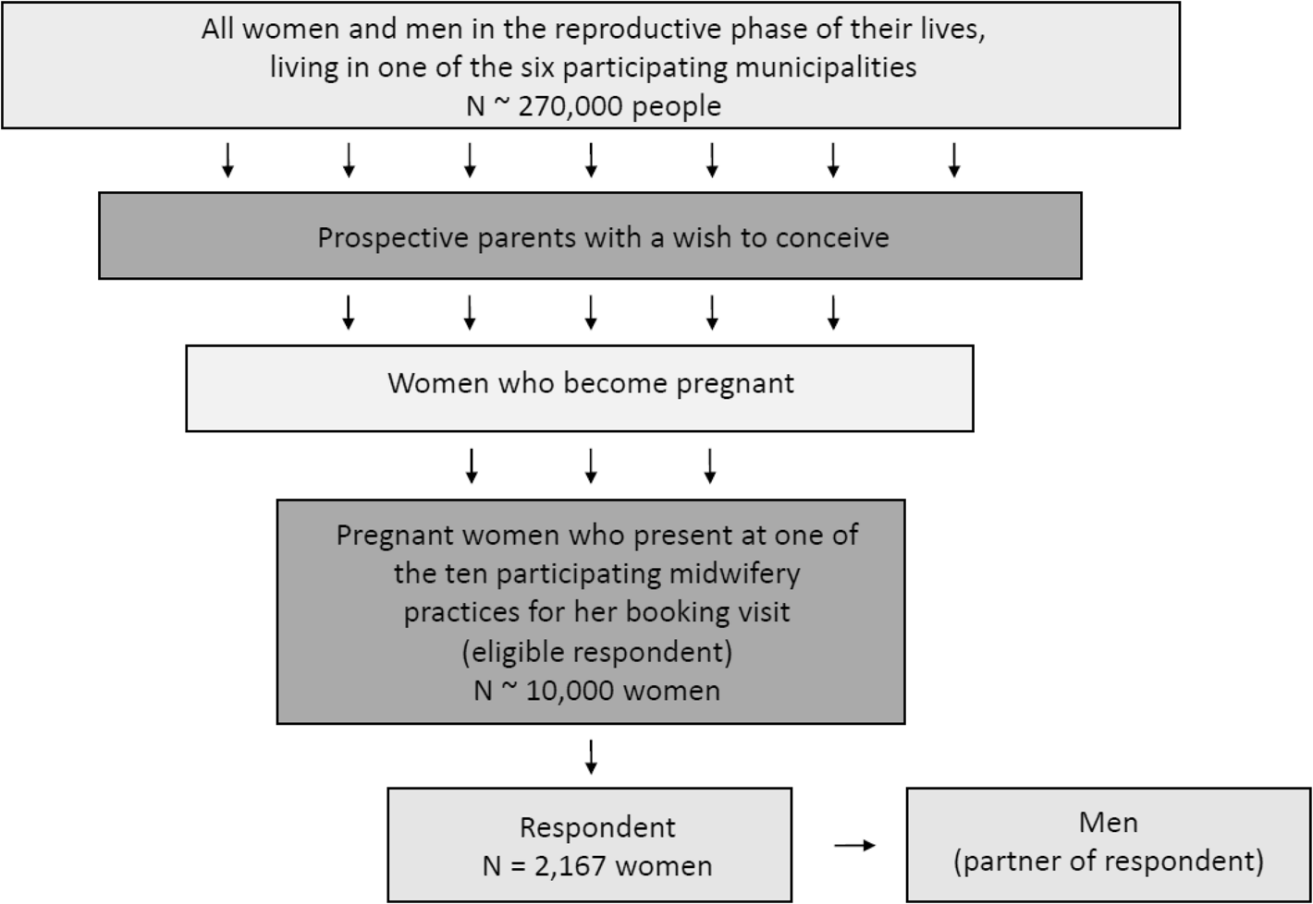
Overview of the studypopulation of the APROPOS-II study. This figure shows how the number of people in the target population results in the total number of respondents for the APROPOS-II study. The intervention targets all men and women in their reproductive life span within a municipality. We aim to include 2267 women in the study

Our intervention targets all men and women in their reproductive life span. In all six participating municipalities together, this is approximately 270,000 people [17]. Figure 2 shows how the number of people in the target population results in the total number of respondents.

### Intervention

The intervention used in this study is a PCC-approach tailored to the needs of prospective parent(s) and healthcare providers in a local setting. This intervention has been developed based on pre-implementation research in the APROPOS feasibility study [13]. The intervention has a dual-track approach based on both the uptake and the provision of PCC.

1. Uptake of PCC: To improve preconceptional lifestyle behaviours and the uptake of PCC, a social marketing strategy, directed towards prospective parent(s), will be distributed in each participating municipality. This online and offline social marketing strategy aims to trigger prospective parents to actively prepare for pregnancy by directing them towards reliable PCC-information and promoting individual PCC-consults.
2. Provision of PCC: To improve interdisciplinary collaboration among healthcare providers, a local care pathway will be developed and implemented in each participating municipality, including interdisciplinary arrangements for collaboration and referral between primary, secondary and tertiary care. Moreover, a working conference will be held among local healthcare providers of multidisciplinary backgrounds and a stakeholder coalition will be formed.

### Implementation of the intervention

**Fig. 3:**
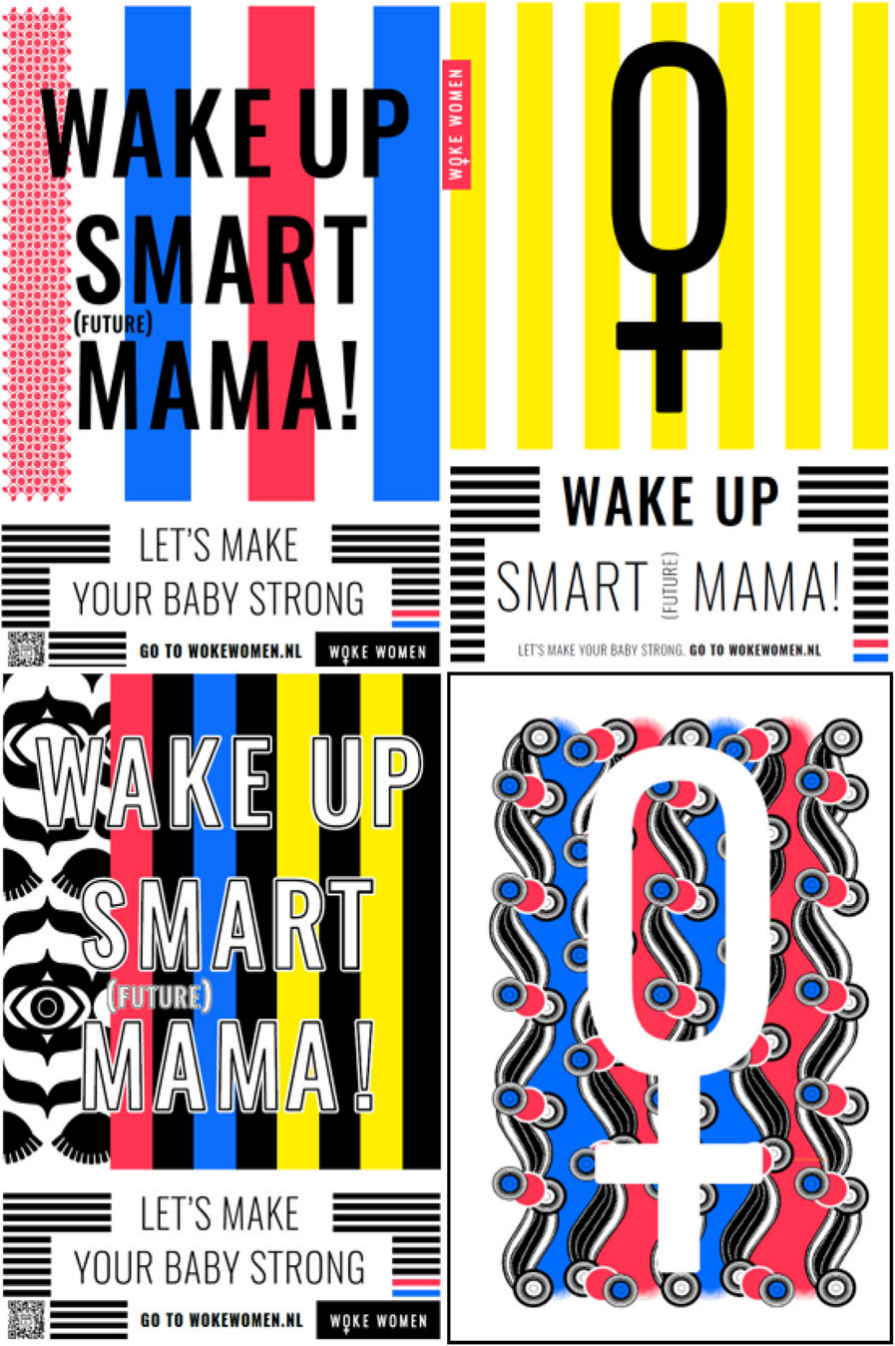
Sociale marketing strategy Woke Women®. Examples of the promotional material (visuals) of the social marketing strategy Woke Women® which has been specifically developed for this study

Concerning the uptake of PCC, the social marketing strategy has been developed by a professional and experienced marketing agency. A social marketing strategy uses commercial marketing technologies to design programs that can influence the behaviour of the target audience in order to improve personal welfare and that of society [18]. The theoretical foundation for this social marketing strategy is based on research regarding the target population’s perception from biological-, emotional-, social- and cultural point of views and resulted in the concept of “Woke Women®”, with the single-minded proposition: “Wake up smart (future) Mama! Let’s make your baby strong”. The social marketing strategy uses different channels of communication, e.g. online marketing, traditional offline marketing channels, word-of-mouth marketing and social networks will be addressed to start a social movement. Its purpose is to use existing social networks to encourage women to motivate each other towards healthy behaviour. Examples of materials used in the social marketing strategy are shown in Fig. 3 and more information about the social marketing strategy can be found on www.wokewomen.nl (in Dutch).

Regarding the provision of PCC, the working conference for the healthcare providers aims to educate them on preconceptional health and risk factors using the national Preconception Indication List (PIL), emphasizes the importance of facilitating PCC and is used to conduct a region-specific bottleneck analysis [5] [19]. Subsequently, a multidisciplinary group of healthcare providers will form a local stakeholder coalition. With the input of these stakeholders, the intervention will be tailored to the participating municipality prior to implementation by adapting the dissemination locations, the design of the intervention and the essential elements included in the intervention.

### Clinical parameters and outcomes

Study parameters will be assessed by a questionnaire distributed among prospective parent(s) by the participating community midwifery practices in the first trimester of pregnancy. The questionnaire is based on validated questionnaires such as WHO - Quality of life questionnaire, the London Measure of Unplanned Pregnancy (LMUP), a validated preconception tool (www.zwangerwijzer.nl) and the APROPOS feasibility study [20, 21]. The questionnaire contains five sections: pregnancy planning, pregnancy preparation, healthy lifestyle behaviours and risk factors, personal situation and the prospective father. There are two versions of the questionnaire, one pre-implementation and one post-implementation. The post-implementation questionnaire contains additional evaluation questions about participant’s experiences with the social marketing strategy. An overview of all the variables in the questionnaire is shown in Table 1. The questionnaire is available in Dutch, English, Polish and Turkish; languages that are mastered by the majority of the inhabitants of the participating municipalities. The questionnaire for prospective fathers is shorter compared to the questionnaire for pregnant women and solely focusses on lifestyle behaviours and health beliefs.

**Table 1:** Definition of primary and secondary outcomes of the APROPOS-II study

	Variable	Definition*
Pregnancy preparation	Level of pregnancy planning	London Measure of Unplanned Pregnancies (LMUP) [21]
	PCC health seeking behaviour	Acquired PCC-information through the internet, books, journals, folders or family & friends.
	PCC-consult	A consultation provided by a healthcare provider.
Modifying lifestyle behaviours & risk factors	Fruit intake	≥ 2 pieces of fruit a day [22]
	Vegetable intake	≥ 200 g of vegetables a day [22]
	Caffeine intake	≤ 1 caffeine-containing beverage [22]
	Exercising	≥ 150 min per week moderate or heavy intensive exercise, spread over various days [23]
	Smoking	No smoking [5, 24]
	Alcohol intake	No alcohol intake [5, 24]
	Folic acid usage	≥400 microgram per day, 4 weeks preconceptionally until 10 weeks gestation [5, 24]
	Medication usage	Risk-free medication [5]
	Psychological distress	A stable emotional state [5]
	Vaccination status	Vaccination status should be discussed with special attention to rubella, measles and whooping cough. Based on individual assessment of antibody titres; (re)vaccinations can be considered [24]
	Working conditions	Avoid contact with harmful working conditions [5]
Prenatal outcomes	Miscarriage	Loss of pregnancy before 24 weeks [25].
	Gestational Diabetes (GDM)	Diagnosed by a 75-g oral glucose tolerance test (OGTT) as the presence of either a fasting glucose level of ≥7.0 mmol/L (126 mg/dl) or a glucose level of ≥7.8 mmol/L (140 mg/dl) after two hours [26, 27]
	Pregnancy-induced hypertension (PIH)	New onset of hypertension (≥ 140 mmHg systolic and/or ≥ 90 mmHg diastolic blood pressure) after 20 weeks gestation measured on at least two occasions four hours apart [28, 29]
	Pre-eclampsia (PE)	PIH accompanied by proteinuria (≥300 mg in 24 h) [29, 30]
	Spontaneous preterm birth	Delivery with spontaneous onset before 37 weeks of gestation [31].
	Referral to secondary care	If complications occur or threaten to occur, the midwife will refer the woman to the obstetrician who will take over the care for as long as deemed necessary [32].
Birth outcomes	Mode of delivery	Spontaneous vaginal delivery, assisted vaginal delivery, or caesarean section [33].
	Referral to secondary care	If complications occur or threaten to occur, the midwife will refer the woman to the obstetrician who will take over the care for as long as deemed necessary [32].
Neonatal outcomes	Small for gestational age (SGA)	Birth weight < 10th percentile, based on Dutch national reference curves adjusted for parity, gestational age, sex and ethnicity [34].
	Congenital anomalies	Structural-morphological, functional and/or biochemical-molecular defects present at birth [35].
	APGAR- score	A score is a sum of the values assigned to the infant at 1 and 5 min of life, with a score of 7 or more indicating that the baby is in good to excellent condition [36].

* *Definitions are based on Dutch standards*

The primary outcome of this study is change in lifestyle behaviours. This is a composite outcome of four modifiable lifestyle behaviours (healthier diet, folic acid use, quit drinking and quit smoking during the preconception period), expressed as the number of women who preconceptionally change at least one of these lifestyle behaviours from inadequate to adequate. The definitions of healthy lifestyle behaviour are based on Dutch guidelines and are provided in Table 1.

Secondary outcomes are pregnancy outcomes (e.g. miscarriage, preterm birth, gestational diabetes) and the uptake of PCC. The definitions of these pregnancy outcomes are given in Table 1 and will be collected from the participants’ medical records after obtaining additional informed consent. The uptake of PCC will be measured through a section in the questionnaire and by the records of the midwifery practices on PCC-consults.

### Implementation parameters and outcomes

The RE-AIM framework will be used to evaluate the intervention and the implementation strategy. This framework is a practical way of evaluating health interventions and has previously been used in studies focused on changing individual behaviours [16]. Regarding patient implementation outcomes, the respondents’ satisfaction towards the PCC-consults will be assessed by elements of the validated ReproQ, which is based on the WHO-concept responsiveness and has been incorporated in our questionnaire [37, 38].

Healthcare providers’ implementation outcomes will be measured by a 23-item validated NoMAD (Normalisation MeAsure Development) questionnaire. The NoMAD questionnaire will be used to describe the health care providers’ views on how the intervention impacts their work and their expectations about whether the intervention could become a routine part of their work [39]. The healthcare providers will receive this questionnaire 3 months before the start of the intervention-phase and a follow-up questionnaire 6 months later. Barriers and facilitators regarding the implementation of the PCC-approach will be collected among healthcare providers and classified using the Flottorp et al. - checklist [40].

Finally, the feasibility of the implementation of the local care pathways will be evaluated among healthcare providers. We will measure to what degree the local care pathway was distributed amongst the healthcare providers and how healthcare providers experienced the convenience of this tool. In each municipality, focus groups with the local stakeholder coalition will be held 9 months after the start of the intervention and will be analysed using thematic analysis to identify key issues and themes. In these focus groups, information will be gathered on how the intervention complies with the heterogeneity of local settings, since differences in healthcare networks, logistics and practical issues call for tailored approaches.

### Sample size calculation

Based on the results from the APROPOS feasibility study, we anticipate on an odds ratio of 1.5 in lifestyle behaviour change during the preconception period (primary outcome). Based on the sample size estimation method of Hemming and Taljaard, with an alpha of 0.05, power of 80% and a low intraclass correlation Coefficient of 0.02, the sample size was calculated to be 363 participants per unit with 6 units (=municipalities; 2167 in total, 23 participants per month per municipality) [41]. Considering our inclusion criteria, almost all women who have their intake visit during the study period are eligible respondents. Based on previous experience, we expect that 50–60% of these women will fill out the questionnaire.

### Statistical analysis

Statistical analysis will be performed using multilevel logistic regression analysis to take into account the clustering of respondents within municipalities. Data will be analysed anonymously on two levels; the respondents’ level (before and after the intervention) and the municipal level (differences between municipalities). Baseline characteristics will be compared between the pre- and post-intervention group and compared in a baseline table. Chi-square analysis and ANCOVA will be used to study the effects of the intervention on the adverse pregnancy outcomes. Adjusted odds ratios will be calculated taking into account the potential confounders: age, educational level and parity. *P*-values < 0.05 are considered statistically significant.

## Discussion

The APROPOS-II study introduces a locally tailored PCC-approach in six municipalities in the Netherlands, that will contribute to current knowledge regarding the implementation and the effectiveness of PCC. Until now there is little data from randomized clinical trials that prove the effectiveness of PCC on maternal and perinatal health outcomes [3, 42, 43]. This evidence is necessary to substantiate the urgency to invest in a comprehensive (inter)national PCC-program. The presented intervention serves the different needs of prospective parents by providing both separate preconception health information and PCC-consultation. This supports the view that most prospective parents will benefit from evidence-based information to prepare themselves for pregnancy, while not every prospective parent will attend a PCC-consult.

Despite the low uptake of PCC-consults, the majority of prospective parents use the internet as their primary source of information regarding preconception health [11]. Women appreciate anonymity and self-management of online information in the privacy of their own home [11, 44]. The aim of the social marketing strategy in this intervention is to trigger women to actively prepare for their pregnancy by guiding them towards a website (www.wokewomen.nl) with practical and evidence-based information to help them improve their preconceptional health status. In addition, we encourage these women when there is a need for extra information to visit a healthcare provider for a PCC-consult.

Previous literature on social marketing strategies showed that it has the potential to improve diet, increase exercise and reduce substances-use like tobacco, and alcohol [18]. Creating a social movement could raise awareness on the importance of PCC and could cause a supportive social environment for preconception health. In addition, research shows that healthcare collaborations in health promotion can result in effective and sustainable benefits for those involved [45]. When effectively facilitated, healthcare collaborations can enable fundamental improvements to community development and health promotion.

Strengths of this stepped-wedge cluster RCT are the diversity of the population in the participating municipalities and the tailored intervention that complies with the heterogeneity of local settings. Moreover, the detailed questionnaire investigating respondents’ lifestyle behaviours, health beliefs and the extent of planning of the current pregnancy based on validated questionnaires can be considered a strength of this study. In addition, the innovative social marketing strategy, the extensive process evaluation and the involvement of the prospective father highlight different and often neglected aspects of PCC-research.

A potential limitation of this study is the occurrence of selection bias, as participants who have actively prepared for their pregnancy are probably more eager to share their experience. However, in our previous feasibility study, response rates were high and the population characteristics were similar before and after introduction of the intervention [13]. Another limitation is the selection strategy, as the respondents of this study are pregnant while the interventions focusses on the preconceptional period. Previous studies already showed that prospective parents are very difficult to identify and recruit, therefore almost all PCC related studies obtain information retrospectively [46]. Finally, active participation of the healthcare providers is crucial to make the local PCC-pathway a success. Fortunately, the healthcare providers involved in the local PCC-pathway are driven to improve the awareness of PCC in their municipality and will be equipped to provide adequate PCC. All community midwifery practices have already been recruited and municipal policy makers are involved throughout the entire project.

With this study, we expect to effectively implement and evaluate a locally tailored PCC-approach. If this intervention will show to improve lifestyle behaviours, leading to improved pregnancy outcomes and future health of prospective parents and their offspring, it may be upscaled to (inter)national implementation.

## Data Availability

Not applicable

## Abbreviations

LMUP: London Measure of Unplanned Pregnancy
NoMAD: Normalisation MeAsure Development
PCC: Preconception Care
RCT: Randomized Controlled Trial
RE-AIM: Reach, Effectiveness, Adoption, Implementation, Maintenance
WHO: World Health Organisation

## References

[1] Atrash HK, Johnson K, Adams M, Cordero JF, Howse J (2006). Preconception care for improving perinatal outcomes: the time to act. Matern Child Health J.

[2] de Weerd S, Steegers EA (2002). The past and present practices and continuing controversies of preconception care. Community Genet.

[3] Temel S, van Voorst SF, Jack BW, Denktaş S, Steegers EAP (2013). Evidence-based preconceptional lifestyle interventions. Epidemiol Rev.

[4] Louis GMB, Cooney MA, Lynch CD, Handal A (2008). Periconception window: advising the pregnancy-planning couple. Fertil Steril.

[5] College Perinatale Zorg (Perinatal Care College). Preconceptie Indicatie Lijst (preconception indication list). 2018. https://www.kennisnetgeboortezorg.nl/wp-content/uploads/2019/06/Preconceptie_Indicatie_Lijst_PIL_.pdf. Accessed 11 June 2019.

[6] Shawe J, Patel D, Joy M, Howden B, Barrett G, Stephenson J (2019). Preparation for fatherhood: a survey of men’s preconception health knowledge and behaviour in England. PLoS One.

[7] Johnson K, Posner SF, Biermann J, Cordero JF, Atrash HK, Parker CS (2006). Recommendations to Improve Preconception Health and Health Care—United States: Report of the CDC/ATSDR Preconception Care Work Group and the Select Panel on Preconception Care. MMWR Recomm Rep.

[8] World health O. meeting to develop a global consensus on preconception care to reduce maternal and childhood mortality and morbidity: World Health Organization headquarters, Geneva, 6–7 February 2012: Meeting report 2013.

[9] M'Hamdi HI, van Voorst SF, Pinxten W, Hilhorst MT, Steegers EA (2017). Barriers in the uptake and delivery of preconception care: exploring the views of care providers. Matern Child Health J.

[10] Poels M, Koster MPH, Boeije HR, Franx A, van Stel HF (2016). Why do women not use preconception care? A systematic review on barriers and facilitators. Obstet Gynecol Surv.

[11] Poels M, Koster MPH, Franx A, van Stel HF (2017). Parental perspectives on the awareness and delivery of preconception care. BMC Pregnancy Childbirth.

[12] Hemsing N, Greaves L, Poole N (2017). Preconception health care interventions: a scoping review. Sex Reprod Healthc.

[13] Poels M, van Stel HF, Franx A, Koster MPH (2018). The effect of a local promotional campaign on preconceptional lifestyle changes and the use of preconception care. Eur J Contracept Reprod Health Care.

[14] Curran GM, Bauer M, Mittman B, Pyne JM, Stetler C (2012). Effectiveness-implementation hybrid designs: combining elements of clinical effectiveness and implementation research to enhance public health impact. Med Care.

[15] Hemming K, Haines TP, Chilton PJ, Girling AJ, Lilford RJ (2015). The stepped wedge cluster randomised trial: rationale, design, analysis, and reporting. Bmj.

[16] King DK, Glasgow RE, Leeman-Castillo B (2010). Reaiming RE-AIM: using the model to plan, implement, and evaluate the effects of environmental change approaches to enhancing population health. Am J Public Health.

[17] Centraal Bureau voor de Statistiek (Central Bureau of Statistics). Kerncijfers Wijken en Buurten (Key figures Districs and Neighborhoods) 2018. [internet] URL: https://www.waarstaatjegemeente.nl [accessed on 06-11-2019].

[18] Gordon R, McDermott L, Stead M, Angus K (2006). The effectiveness of social marketing interventions for health improvement: what's the evidence?. Public Health.

[19] Poels M, Koster MP, Franx A, van Stel HF (2017). Healthcare providers' views on the delivery of preconception care in a local community setting in the Netherlands. BMC Health Serv Res.

[20] Group TW (1998). The World Health Organization quality of life assessment (WHOQOL): development and general psychometric properties. Soc Sci Med.

[21] Barrett G, Smith SC, Wellings K (2004). Conceptualisation, development, and evaluation of a measure of unplanned pregnancy. J Epidemiol Community Health.

[22] The Netherlands Nutrition Centre (het voedingscentrum). 2015. Zwangerschap (Pregnancy) [internet] URL: https://www.voedingscentrum.nl/professionals/kindervoeding-0-4-jaar/zwangerschap.aspx [].

[23] Gezondheidsraad (Health Council). Bewegingsrichtlijn (Movement Directive) 2017. 2017;2017/08. .

[24] Shawe J, Delbaere I, Ekstrand M, Hegaard HK, Larsson M, Mastroiacovo P (2015). Preconception care policy, guidelines, recommendations and services across six European countries: Belgium (Flanders), Denmark, Italy, the Netherlands, Sweden and the United Kingdom. Eur J Contracept Reprod Health Care.

[25] Royal College of O, Gynaecologists. The management of early pregnancy loss. Green top Guideline. 2006(25).

[26] Alberti KGMM, Zimmet PZ (1998). Definition, diagnosis and classification of diabetes mellitus and its complications. Part 1: diagnosis and classification of diabetes mellitus. Provisional report of a WHO consultation. Diabet Med.

[27] NVOG: Dutch Society of Obstetrics and Gynecology. Diabetes mellitus en zwangerschap. (Diabetes mellitus and pregnancy) 2010;Version 2.0. [internet] URL: https://www.nvog.nl/wp-content/uploads/2018/02/Diabetes-mellitus-en-zwangerschap-2.0-04-06-2010.pdf [accessed 2019-07-12].

[28] Brown Mark A., Lindheimer Marshall D., de Swiet Michael, Assche Andre Van, Moutquin Jean-Marie (2001). The Classification and Diagnosis of the Hypertensive Disorders of Pregnancy: Statement from the International Society for the Study of Hypertension in Pregnancy (ISSHP). Hypertension in Pregnancy.

[29] NVOG: Dutch Society of Obstetrics and Gynecology. Chronische Hypertensie in de zwangerschap (Chronic hypertension in pregnancy) - Guideline. 2011. [internet] URL: https://www.nvog.nl/wp-content/uploads/2017/12/Chronische-hypertensie-in-de-zwangerschap-2.0-16-03-2005.pdf [accessed 2019-07-12].

[30] Uzan J, Carbonnel M, Piconne O, Asmar R, Ayoubi JM. Pre-eclampsia: pathophysiology, diagnosis, and management. Vasc Health Risk Manag. 2011;7:467–74. 10.2147/VHRM.S20181 PMC3148420 21822394

[31] Lumley J (2003). Defining the problem: the epidemiology of preterm birth. BJOG Int J Obstet Gynaecol.

[32] Amelink-Verburg MP, Buitendijk SE (2010). Pregnancy and labour in the Dutch maternity care system: what is normal? The role division between midwives and obstetricians. J Midwifery Women’s Health.

[33] Wu J, Viguera A, Riley L, Cohen L, Ecker J (2002). Mood disturbance in pregnancy and the mode of delivery. Am J Obstet Gynecol.

[34] Visser GHA, Eilers PHC, Elferink-Stinkens PM, Merkus HMWM, Wit JM (2009). New Dutch reference curves for birthweight by gestational age. Early Hum Dev.

[35] Czeizel AE, Intody Z, Modell B (1993). What proportion of congenital abnormalities can be prevented?. Bmj.

[36] Leuthner SR, Das UG (2004). Low Apgar scores and the definition of birth asphyxia. Pediatr Clin N Am.

[37] Scheerhagen M, Van Stel HF, Birnie E, Franx A, Bonsel GJ (2015). Measuring client experiences in maternity care under change: development of a questionnaire based on the WHO responsiveness model. PLoS One.

[38] Valentine NB, de Silva A, Kawabata K, Darby C, Murray CJL, Evans DB (2003). Health system responsiveness: concepts, domains and operationalization. Health systems performance assessment: debates, methods and empiricism.

[39] May CR, Mair FS, Dowrick CF, Finch TL (2007). Process evaluation for complex interventions in primary care: understanding trials using the normalization process model. BMC Fam Pract.

[40] Flottorp SA, Oxman AD, Krause J, Musila NR, Wensing M, Godycki-Cwirko M (2013). A checklist for identifying determinants of practice: a systematic review and synthesis of frameworks and taxonomies of factors that prevent or enable improvements in healthcare professional practice. Implement Sci.

[41] Hemming K, Taljaard M (2016). Sample size calculations for stepped wedge and cluster randomised trials: a unified approach. J Clin Epidemiol.

[42] Hussein N, Kai J, Qureshi N (2016). The effects of preconception interventions on improving reproductive health and pregnancy outcomes in primary care: a systematic review. Eur J Gen Pract.

[43] Whitworth M, Dowswell T. Routine pre-pregnancy health promotion for improving pregnancy outcomes. Cochrane Database Syst Rev. 2009;4. 10.1002/14651858.CD007536.pub2 PMC4164828 19821424

[44] van Dijk MR, Oostingh EC, Koster MPH, Willemsen SP, Laven JSE, Steegers-Theunissen RPM (2017). The use of the mHealth program smarter pregnancy in preconception care: rationale, study design and data collection of a randomized controlled trial. BMC pregnancy childbirth.

[45] Estacio Emee, Oliver Mike, Downing Beth, Kurth Judy, Protheroe Joanne (2017). Effective Partnership in Community-Based Health Promotion: Lessons from the Health Literacy Partnership. International Journal of Environmental Research and Public Health.

[46] Gollenberg AL, Mumford SL, Cooney MA, Sundaram R, Louis GM (2011). Validity of retrospectively reported behaviors during the periconception window. J Reprod Med.

